# Predictors of Anti-TNF Therapy Failure among Inflammatory Bowel Disease (IBD) Patients in Saudi Arabia: A Single-Center Study

**DOI:** 10.3390/jcm11144157

**Published:** 2022-07-18

**Authors:** Othman Alharbi, Abdulrahman M. Aljebreen, Nahla A. Azzam, Majid A. Almadi, Maria Saeed, Baraa HajkhderMullaissa, Hassan Asiri, Abdullah Almutairi, Yazed AlRuthia

**Affiliations:** 1 Gastroenterology Division, Department of Medicine, King Saud University Medical City, King Saud University, Riyadh 11451, Saudi Arabia; amaljebreen@gmail.com (A.M.A.); nahla5_99@yahoo.com (N.A.A.); majid.almadi@gmail.com (M.A.A.); mariasaeed@live.com (M.S.); b.hajkhder@windowslive.com (B.H.); 2 Division of Gastroenterology, The McGill University Health Center, Montreal General Hospital, McGill University, Montreal, QC H3A 0G4, Canada; 3 Department of Clinical Pharmacy, College of Pharmacy, King Saud University, Riyadh 11451, Saudi Arabia; hassan.139468@gmail.com (H.A.); mr.3bo198od@gmail.com (A.A.); 4 Pharmacoeconomics Research Unit, Department of Clinical Pharmacy, College of Pharmacy, King Saud University, Riyadh 11451, Saudi Arabia

**Keywords:** inflammatory bowel disease, infliximab, adalimumab, Crohn’s disease, ulcerative colitis, biologic failure, Saudi Arabia

## Abstract

Background: The advent of monoclonal antibodies (mAbs) has revolutionized the management of many immune-mediated diseases such as inflammatory bowel disease (IBD). Infliximab and adalimumab were the first mAbs approved for the management of IBD, and are still commonly prescribed for the treatment of both Crohn’s disease (CD) and ulcerative colitis (UC). Although mAbs have demonstrated high effectiveness rates in the management of IBD, some patients fail to respond adequately to mAbs, resulting in disease progression and the flare-up of symptoms. Objective: The objective was to explore the predictors of treatment failure among IBD patients on infliximab (INF) and adalimumab (ADA)—as demonstrated via colonoscopy with a simple endoscopic score (SES–CD) of ≥1 for CD and a Mayo score of ≥2 for UC—and compare the rates of treatment failure among patients on those two mAbs. Methods: This was a prospective cohort study among IBD patients aged 18 years and above who had not had any exposure to mAbs before. Those patients were followed after the initiation of biologic treatment with either INF or ADA until they were switched to another treatment due to failure of these mAbs in preventing the disease progression. Univariate and multiple logistic regressions were conducted to examine the predictors and rates of treatment failure. Results: A total of 146 IBD patients (118 patients on INF and 28 on ADA) met the inclusion criteria and were included in the analysis. The mean age of the patients was 31 years, and most of them were males (59%) with CD (75%). About 27% and 26% of the patients had penetrating and non-stricturing–non-penetrating CD behavior, respectively. Patients with UC had significantly higher odds of treatment failure compared to their counterparts with CD (OR = 2.58, 95% CI [1.06–6.26], *p* = 0.035). Those with left-sided disease had significantly higher odds of treatment failure (OR = 4.28, 95% CI [1.42–12.81], *p* = 0.0094). Patients on ADA had higher odds of treatment failure in comparison to those on INF (OR = 26.91, 95% CI [7.75–93.39], *p* = 0.0001). Conclusion: Infliximab was shown to be more effective in the management of IBD, with lower incidence rates of treatment failure in comparison to adalimumab.

## 1. Introduction

Inflammatory bowel disease (IBD) is a universal term for chronic or relapsing inflammation of the gastrointestinal tract and generally refers to two autoimmune disorders: ulcerative colitis (UC) and Crohn’s disease (CD) [1,2]. Multiple risk factors are associated with higher incidence of IBD, such as urban residence, exposure to antibiotics, use of oral contraceptives, consumption of soft drinks, vitamin D deficiency, and appendectomy [3,4]. Although the highest incidence and prevalence rates of IBD were reported in western countries [5,6,7,8,9,10], the incidence and prevalence rates of IBD are also increasing in other parts of the world, particularly in newly industrialized countries such as India, Brazil, China, and Taiwan [5,6]. In Saudi Arabia, the incidence and prevalence rates of IBD are largely unknown. However, a few studies have reported an annual incidence rate of CD that ranges between 1.46 and 1.66 per 100,000 people for CD, and of 2.33 per 100,000 people for UC [11,12]. The majority of CD patients in Saudi Arabia are young (17–40 years of age), with a significant proportion of them (e.g., 49%) having ileocolonic involvement and non-stricturing–non-penetrating disease behavior [13], while the majority of UC patients are male, young, and have extensive UC (43%) or left-sided colitis (35%) [14].

Pharmacotherapy is the mainstay of IBD treatment, and can be classified into two groups of therapies: biologic—such as Anti-tumor necrosis factor (anti-TNF) agents (e.g., infliximab (INF) and adalimumab (ADA)); anti-interleukin (IL)-12 and IL-23 (e.g., ustekinumab); and anti-integrin (e.g., vedolizumab) [15,16]—and non-biologic (e.g., mesalamine, sulfasalazine, prednisolone, methylprednisolone, 6-mercaptopurine, azathioprine, methotrexate, etc.) [17]. The management of IBD has evolved over time from the alleviation of symptoms and improving patients’ quality of life to mucosal healing [18]. However, about one-third of IBD patients on biologics fail to respond to therapy by showing no improvement in mucosal healing [19]. These are considered primary non-responders (PNR), and they usually fail to respond to biologic therapies during the induction phase (8–12 weeks) [20]. Additionally, 23% to 46% of IBD patients on biologics experience treatment relapse after the initial response, and they are usually referred to as secondary non-responders [20].

The failure of biologic therapies among IBD patients has been linked to several factors, such as long disease duration (e.g., >2 years), disease behavior and phenotype, smoking, C-reactive protein (CRP) levels, disease severity, albumin levels, and cytokine expression. [20,21,22] Additionally, patients with a history of anti-TNF therapy failure are less likely to respond to second-line anti-TNF biologics [23,24,25] Moreover, older adults (e.g., ≥65 years) with IBD who were started on anti-TNF (INF or ADA) agents showed a higher treatment-failure rate [26,27]. In contrast, the concomitant use of anti-TNF biologics (INF or ADA) with an immunomodulator, such as azathioprine, and proactive therapeutic drug monitoring are associated with lower risk of treatment failure with anti-TNF therapy [28,29]. In Saudi Arabia, the utilization of biologics in general, and anti-TNF, in particular, is believed to be as high as 60% among CD patients [30,31]. However, no study has, so far, compared the rates of treatment failure among IBD patients treated with INF or ADA, which are the most-commonly utilized biologics in Saudi Arabia for IBD treatment [31]. Therefore, the aims of this study were to compare the rates and predictors of treatment failure among IBD patients on anti-TNF biologics in Saudi Arabia, as demonstrated by the presence of deep ulcers and a simple endoscopic score (SES–CD) of one or more for CD, and a Mayo score of two or more for UC patients [32,33].

## 2. Methods

### 2.1. Study Design and Population

This was a single-center, prospective, registry-based cohort study in a university-affiliated tertiary-care referral center in Riyadh, Saudi Arabia, with approximately 1200 staffed beds. All IBD patients were enrolled in an electronic registry that was established in 2015. Patients’ sociodemographic characteristics (e.g., age, gender, city, geographic region, nationality) and medical characteristics (e.g., type of IBD (UC vs. CD), disease behavior, disease severity, location of the lesion, duration of illness, prescribed medications, etc.) were prospectively collected for all registered patients.

In this study, adult patients (≥18 years) with IBD who were biologic-naïve prior to anti-TNF therapy were included in this study. Those patients were followed prospectively from the time of anti-TNF biologic treatment the initiation to failure, which was defined as disease progression; this was demonstrated by the colonoscopy that led to discontinuation of the anti-TNF biologic (INF or ADA) and switching to another anti-TNF or other biologics. Patients under 18 years of age, those with previous exposure to biologic therapy, patients with missing observations, and those who were followed up for less than 12 months were excluded from the study. The study sample was followed from May 2015 to September 2021.

### 2.2. Ethical Approval of the Study

The study was approved by the Institutional Review Board of the King Saud University College of Medicine, Riyadh, Saudi Arabia (Project No. E-11-538).

### 2.3. Statistical Analysis

The minimum sample size was estimated to be 113 patients for multiple logistic regression with seven predictor variables, whereby the dependent variable was defined as treatment failure, and an odds ratio (OR) of 2 in favor of INF was used as an effect size (β = 0.2, α = 0.05, and power of 80%). Descriptive statistics using frequencies, percentages, means, and standard deviations were used to present the baseline characteristics of the study sample. A chi-square test, Fisher’s exact test, and Student’s t-test were performed to compare patient characteristics across the INF and ADA groups. Univariate logistic regressions were conducted to examine the individual relationships between the use of ADA versus INF, age, gender, UC versus CD, location of lesions (e.g., terminal ileum, ileocolon, pancolitis, etc.), disease behavior (e.g., extensive, penetrating, stricturing, etc.), disease duration in years, and treatment failure. Multiple logistic regression was conducted to examine the odds of treatment failure for ADA versus INF, controlling for age, gender, disease behavior, disease duration, type of IBD (UC versus CD), and location of lesions. These variables were selected based on the previously published studies that showed potential relationships between them and the rates of treatment failure among IBD patients on biologics [19,21,27].

## 3. Results

### 3.1. Patient Baseline Characteristics

**Figure 1:**
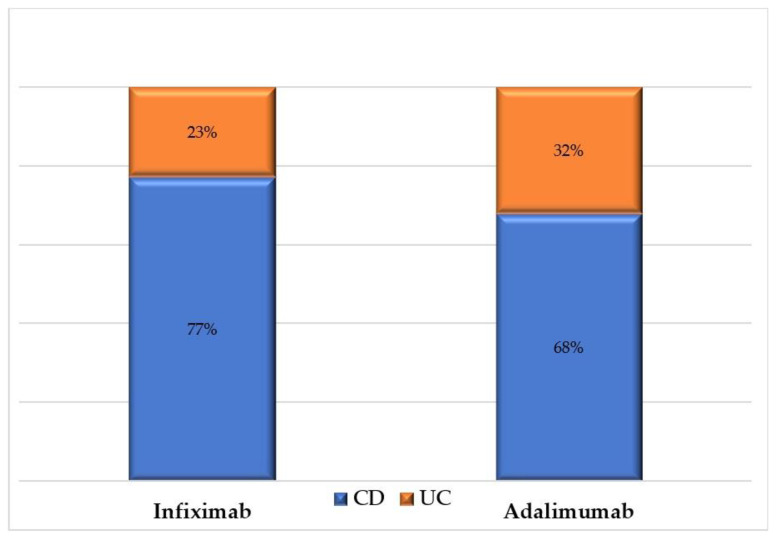
Percentage of patients with ulcerative colitis (UC) and Crohn’s disease (CD) among patients on infliximab and adalimumab.

Out of the 202 patients’ records that were reviewed for inclusion, 146 patients (118 patients on INF and 28 patients on ADA) met the inclusion criteria and were included in the study. The mean age of the patients was 31 years, with no significant difference between the two treatment groups (INF vs. ADA). Most of the patients on INF were males (62.71%), while most of the patients on ADA were females (57.14%). Patients on ADA had, on average, a disease duration two years longer than their counterparts on INF (7.75 vs. 5.67 years, *p*-value = 0.03). More than two-thirds of patients had CD (75.34%), as shown in Figure 1, and the ileocolonic region was the main affected area in 50% of patients. Most patients had non-stricturing–non-penetrating CD (26.39%) or penetrating (27.1%) CD behavior. The patients’ baseline characteristics are shown Table 1. All patients were on immunosuppressants such azathioprine (79.54%) and methotrexate (20.46%).

### 3.2. The Rates and Predictors of Treatment Failure for Infliximab (INF) and Adalimumab (ADA)

**Figure 2:**
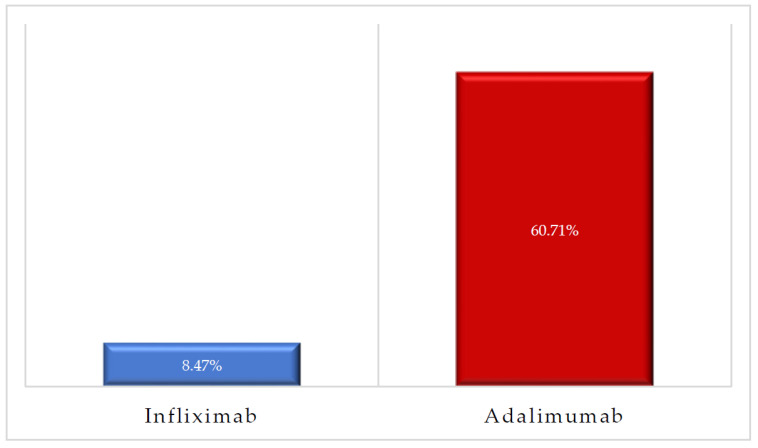
The rates of treatment failure among patients treated with infliximab and adalimumab.

About 61% of patients on ADA had treatment failure in comparison to 8.47% of patients on INF as shown in Figure 2. Out of the 91 CD patients on INF, only four patients (4.39%) failed the treatment and were switched to another biologic such as certolizumab and ustekinumab, while 11 patients (57.89%) out of the 19 CD patients on ADA failed the treatment. A total of 5 patients (18.52%) out of 27 UC patients on INF failed the treatment and were switched to other biologics such as vedolizumab, while 6 patients (66.67%) out of 9 UC patients on ADA failed the treatment and were switched to INF or vedolizumab. Furthermore, patients on ADA had more than 16 times higher odds of treatment failure in comparison to their counterparts on INF (OR = 16.69, 95% CI [6.165–45.25], *p* < 0.0001) as shown in Table 2.

In addition, patients with UC had more than 2.5 times higher odds of treatment failure in comparison to their counterparts with CD (OR = 2.585, 95% CI [1.066–6.266], *p* = 0.0355). Moreover, the left-sided location of lesions for UC patients was associated with more than four times higher odds of treatment failure than their counterparts with other affected locations (OR = 4.278, 95% CI [1.429–12.808], *p* = 0.0094). The odds of treatment failure among patients on ADA versus INF stayed significant (OR = 26.91, 95% CI [7.75–93.39], *p* = 0.0001), even after controlling for age, gender, type of IBD (UC vs. CD), disease location for UC patients and behavior for CD patients, and duration of illness, as shown in Table 3.

## 4. Discussion

In this study the predictors and rates of treatment failure among a cohort of 146 biologic-naïve IBD patients on anti-TNF agents (INF and ADA) were explored. The use of INF was associated with significantly lower odds of treatment failure in comparison to ADA. Moreover, ulcerative colitis, and particularly left-sided UC, was associated with higher odds of treatment failure in comparison to CD. However, this difference in the rate of treatment failure among UC patients did not become significant after controlling for age, gender, location of lesions, and duration of illness. The importance of this study stems from the fact that each ethnic population has a different phenotype of UC or CD, which results in different treatment responses [34]. For example, no difference in treatment outcomes was observed between ADA and INF among a sample of 113 biologic-naïve UC patients in South Korea after a follow-up period of five years according to Lee et al. [34,35]. Similarly, another prospective cohort study that included CD patients between 2007 and 2011 in New Zealand and Australia found that INF and ADA had similar treatment response rates [36]. However, in another nationwide registry-based study that compared the all-cause hospitalization among biologic-naïve UC patients in Denmark who were treated with INF and ADA, the risk of hospitalization was almost two times higher among patients treated with ADA in comparison to their counterparts who were treated with INF [37]. However, ADA had the highest persistence and lowest switching rates among both CD and UC patients according to a retrospective cohort study that utilized private insurance claims data in the United States [38]. Therefore, the findings of our study, which showed a significantly higher rate of treatment failure among IBD patients on ADA in comparison to INF, prove that IBD patients from different nationalities or ethnic groups respond differently to anti-TNF agents.

Unlike previously published studies which showed that older age (e.g., ≥60 years) was correlated with higher treatment failure rates and serious infections among IBD patients on anti-TNF agents [26,27,39], no significant relationship was found between treatment failure and age in this study. This might be due to the small sample size as well as the younger patient population, with a mean age of 24 years. Similarly, disease duration was not associated with higher or lower rates of treatment failure among IBD patients on anti-TNF agents (INF and ADA), unlike other studies that suggested higher rates of treatment failure among those with long disease durations [20]. However, in a single-center study that examined the role of disease duration on biologic treatment failure among a sample of 160 UC patients in the United States, short disease duration was associated with higher rates of treatment failure [40]. Therefore, the role that disease duration plays in the rates of treatment failure among IBD biologic-naïve patients remains uncertain [41]. Female gender was not associated with higher or lower rates of treatment failure, despite some evidence that suggests poorer subjective symptoms among women in comparison to their male counterparts [42]. However, since this study used an objective measure to demonstrate treatment failure (e.g., colonoscopy), no difference was found in the rates of treatment failure, which is in line with the preponderance of evidence [42]. The rates of treatment failure among UC patients were found to be higher in comparison to their CD counterparts. Although the rates of biologic failure among UC and CD patients on anti-TNF agents were not found to be different in the preponderance of evidence [43], some studies have suggested higher rates of hospitalization and treatment failure among UC patients on ADA in comparison to their counterparts on INF [37]. Although some studies suggest a potential role of CD behavior in the rates of treatment failure [21], this relationship is controversial and was not confirmed in this study. Disease localization, such as left-sided UC, was associated with a higher risk of treatment failure, but this relationship disappeared after controlling for several confounders in the multiple logistic regression.

Although this is the first study, to the best of our knowledge, to examine the rates of anti-TNF biologics’ treatment failure and factors associated with the treatment failure among biologic-naïve adult IBD patients in a Middle-Eastern population, it has multiple limitations that must be acknowledged. First, this is a single-center study with a relatively small sample size, particularly among those on ADA, which limits the generalizability of the study findings. The small sample size of patients on ADA was mainly due to physician preference to start their patients on INF, as well as budget constraints, since the acquisition cost of ADA is significantly higher than that of infliximab. Moreover, some patients prefer intravenous administration every eight weeks at the hospital over subcutaneous administration every two weeks. Secondly, this is an observational study with non-randomized sampling and diminished internal validity, such as the disproportionate number of patients in each treatment arm, as well as the number of patients with CD and UC. Thirdly, not all confounders were controlled for, such as the use of corticosteroids, which might have impacted the results due to the missing observations. Additionally, the side effects of these two mAbs (INF & ADA) were not captured in the collected data.

## 5. Conclusions

The findings of this study highlight the differences in IBD patients’ responses to anti-TNF biologics and rates of treatment failure among different ethnicities, which can be related to different IBD phenotypes. INF showed significantly lower rates of treatment failure among biologic-naïve adult IBD patients in comparison to ADA. However, these results should be substantiated in future studies with larger sample sizes and more robust study designs.

## Figures and Tables

**Table 1:** Patient baseline characteristics.

Characteristic	ADA (*n*= 28)	INF (*n*= 118)	*p*-Value	Total = 146
**Age (years), mean ± SD**	32.86 (11.54)	30.77 (9.91)	0.3339	31.17 (10.23)
Gender				
Male, *n* (%)	12 (42.86%)	74 (62.71%)	0.055	86 (58.90%)
Female, *n* (%)	16 (57.14%)	44 (37.29%)		60 (41.09%)
Age at diagnosis (years), mean ± SD	24.07 (11.09)	24.26 (8.49)	0.9199	24.22 (9.005)
Disease duration (years), mean ± SD	7.75 (4.88)	5.67 (4.55)	0.0337	6.07 (4.67)
Diagnosis				
Crohn’s disease, *n* (%)	19 (67.86%)	91 (77.12%)	0.307	110 (75.34%)
Ulcerative colitis, *n* (%)	9 (32.14%)	27 (22.88%)		36 (24.66%)
Location ^†^				
Terminal ileum, *n* (%)	5 (17.86%)	28 (24.14%)	0.1056	33(22.92%)
Colon, *n* (%)	0 (0%)	2 (1.72%)		2(1.39%)
Ileocolon, *n* (%)	14 (50%)	58 (50%)		72(50%)
Pancolitis, *n* (%)	0 (0%)	9 (7.76%)		9(6.25%)
Left-sided, *n* (%)	7 (25%)	9 (7.76%)		16(11.11 %)
Proctitis, *n* (%)	0 (0%)	6 (5.17%)		6(4.17%)
Extensive, *n* (%)	2 (7.14%)	4 (3.45%)		6(4.17%)
Behavior ^†^				
Stricturing, *n* (%)	5 (17.86%)	21 (18.1%)	0.886	26 (18.06%)
Non-stricturing–non-penetrating, *n* (%)	5 (17.86%)	33 (28.45%)		38 (26.39%)
Penetrating, *n* (%)	8 (28.57%)	31 (26.72%)		39 (27.08%)
Severe, *n* (%)	4 (14.29%)	11 (9.48%)		15 (10.42%)
Moderate, *n* (%)	4 (14.29%)	13 (11.21%)		17 (11.81%)
Remission, *n* (%)	1 (3.57%)	5 (4.31%)		6 (4.17%)
Mild, *n* (%)	0 (0%)	2 (1.72%)		2 (1.39%)
Smoking Status ^†^				
Smoker, *n* (%)	3 (10.71%)	10 (8.55%)	0.6575	13(8.96%)
Non-smoker, *n* (%)	24 (85.71%)	104 (88.89%)		128(88.27%)
Ex-smoker, *n* (%)	1 (3.57%)	3 (2.56%)		4(2.77%)
**ALB ^†^, mean ± SD**	35.58 (3.52%)	38.23 (22.41%)	0.2378	37.77 ± 20.39
**HB ^†^, mean ± SD**	129.4 (19.86)	128.3 (17.40)	0.7958	128.57 ± 17.78
**WBC ^†^, mean ± SD**	7.07 (2.52)	7.35 (2.33)	0.6081	7.30 ± 2.36
**CRP ^†^, mean ± SD**	8.36 (9.51)	13.06 (21.05)	0.0939	12.24 ± 19.58

**^†^** Behavior and location are missing in two patients taking INF; smoking status is missing in one patient taking INF; average ALB is missing in four patients on ADA and six patients on INF.

**Table 2:** Univariate logistic regression for potential predictors of biologic treatment failure.

Variable	Odds Ratio (OR)	95% Confidence Limits (CI)		*p*-Value
Adalimumab vs. infliximab	16.69	6.16	45.25	<0.0001
Age	1.01	0.97	1.05	0.582
Female gender	0.81	0.34	1.92	0.635
**UC vs. CD**	**2.56**	**1.07**	**6.27**	**0.036**
Terminal ileum	0.37	0.10	1.32	0.126
Ileocolon	0.94	0.41	2.18	0.893
Pancolitis	2.35	0.55	10.08	0.249
**Left-sided**	**4.27**	**1.43**	**12.81**	**0.009**
**Disease behavior**				
Extensive	0.87	0.10	7.83	0.906
Penetrating	1.19	0.48	3.01	0.705
Stricturing	0.52	0.145	1.88	0.321
Non-stricturing Non-penetrating	0.59	0.21	1.69	0.329
Disease duration	1.03	0.95	1.13	0.461

**Table 3:** Multiple logistic regression for the association between treatment failure and the utilization of adalimumab vs. infliximab.

Variable	Odds Ratio (OR)	95% Confidence Limits (CI)		*p*-Value
Adalimumab vs. infliximab	**26.91**	**7.75**	**93.39**	**0.0001**
**Age**	0.99	0.94	1.05	0.825
**Female gender**	0.26	0.072	0.93	0.380
UC vs. CD	**2.34**	**0.54**	**10.14**	**0.256**
Left-sided	**1.89**	**0.28**	**12.34**	**0.508**
**Stricturing**	0.49	0.10	2.56	0.403
**Disease duration**	0.97	0.86	1.11	0.728

## Data Availability

The data are available upon request to the corresponding author (Y.A.).
